# Individual Strivings in Social Comparison Processes: Achievement Motivation Goals in the Big-Fish-Little-Pond Effect

**DOI:** 10.3389/fpsyg.2022.677997

**Published:** 2022-04-18

**Authors:** Alessandra Cecalupo, Mara Marini, Federica Scarci, Stefano Livi

**Affiliations:** Department of Developmental and Social Psychology, University of Rome “La Sapienza”, Rome, Italy

**Keywords:** big-fish-little-pond effect (BFLPE), achievement goal theory, social comparison, future expectations, upper secondary education (high school)

## Abstract

In school settings, adolescents recur to different sources of information to create their beliefs about future possibilities. Social comparison processes and personal goals related to achievement play an important role in shaping these beliefs. Drawing upon literature concerning the Big-Fish-Little-Pond effect and the Achievement Goal Theory, the present study aimed at understanding how adolescents attending the last year of secondary school (*n* = 689; *M*_age_ = 18.15; *SD* = 0.57) perceive their possibilities of potentially having a better future than their classmates. In particular, we sought to understand in what way this perception is influenced by students’ perceived relative position in their class—which accounts for the social comparison process—and its interaction with different types of achievement goals (mastery-approach goals, mastery-avoidance goals, performance-approach goals, and performance-avoidance goals). Results showed that perceived relative position mediated the relationship between the predictors (classmates’ average achievement and individual achievement) and future expectations. Furthermore, analyses of moderated mediation showed that both performance-approach and performance-avoidance goals reduced the impact of a low perceived relative position on future expectations, while mastery-approach and mastery-avoidance goals did not moderate its effect.

## Introduction

### The Big-Fish-Little-Pond Effect

The Big-Fish-Little-Pond effect (BFLPE) was theorized by Marsh and Parker (1984) as an application of the Social Comparison theory (Festinger, 1954) in school settings. The fundamental idea underlying their theorizations refers to the notion that students engage in social comparison processes with their schoolmates whenever they are trying to form their academic self-concept (Marsh and Parker, 1984), or they are generally trying to evaluate their academic abilities. This concept implies that students do not judge themselves only based on their grades, but they also actively search for a frame of reference to form an accurate idea of themselves, and they find this frame of reference in their peers’ average academic achievement. Hence, self-concept can be considered as a multidimensional construct affected by its relationships with different and various influences. Marsh has particularly stressed the multidimensionality of self-concept and has tried, over the years, to widen the existent literature regarding this construct (e.g., Marsh, 1990, 1993; Marsh et al., 1988). Starting from these assumptions, it was theorized that equally able students end up having different views of their achievements depending on how high their peers’ average level is (Marsh and Parker, 1984; Marsh, 1987). Therefore, a student who attends a higher achieving school tends to have a lower concept of her/his academic achievement and school abilities, whereas a student who is embedded in a lower-achieving educational context ends up having a higher academic self-concept when comparing herself/himself to other students. Based on these hypotheses and on the results that the corpus of studies on the BFLPE has shown, it could be concluded that it is better to be a “big fish in a little pond” than a “little fish in a big pond.” This means that, for a student, attending schools or educational programs in which the average achievement is lower or, at least, not higher than her/his own could be better. As explained later on, this assumption could be considered reductive. Some other factors might influence the relationship between social comparison, self-evaluation, and resulting academic outcomes.

The important implications of engaging in social comparison processes in educational settings were already the focus of the Relative Deprivation theory [RD, see Walker and Pettigrew (1984), for an overview of the theme] and, in particular, of Davis’s application of this theory in educational settings (Davis, 1966). In fact, as Davis had already pointed out with his “campus-as-a-frog-pond” paradigm, aspects related to academic life can have an important impact on students’ decisions and choices regarding their future. In particular, his research focused on individual perceptions of relative deprivation that develop consequently to engaging in social comparisons with fellow college students. While Davis’s research mainly focused on college students, in some of Marsh and colleagues’ studies (e.g., Marsh and Yeung, 1997; Marsh et al., 2007; Nagengast and Marsh, 2012; Parker et al., 2014) the samples consisted of younger students. Those studies showed how social comparison processes influence students’ views of their future self and self-worth. They also showed how, even at younger ages, decisions regarding future courses and academic paths largely depend on how students evaluate themselves depending on their peers’ achievements.

In 2008, Dai and Rinn stated that studies about the BFLPE conducted until then showed several limitations. Firstly, they stated that numerous studies had never actually analyzed possible moderators or mediators of the impact that social comparison has on students’ academic self-concept and other dimensions related to it (Dai and Rinn, 2008). They suggested that the BFLPE, especially in early studies, was considered generally valid and independent either from psychological variables or from situational influences, such as the role of perceptions, current motives, environments, and contexts. They also pointed out that the real limitation of numerous works about the BFLPE lied, primarily, in *assuming* that certain variables mediated or moderated the impact of peers’ achievement on self-concept and other constructs, an assumption that was not necessarily based on empirical findings. Moreover, as Dai and Rinn (2008) pointed out, social comparison is made up of dynamic processes that change and evolve over time because of development, aging, and cognitive maturation, and because of the active role that the individual—in conformity with current perceptions, motives, needs, and goals—plays in them. Generally speaking, the studies that analyzed the limitations in the BFLPE showed the necessity of deepening our understanding of the effect by considering the role of other variables that might change or better explain the effect of social comparison processes on self-concepts and self-construals (see also Pomerantz et al., 1995; Huguet et al., 2001).

Recent studies tried to take this issue into account to overcome said limitations (e.g., Seaton, 2007; Seaton et al., 2010, 2011; Marsh et al., 2021). Huguet et al. (2009) and Thijs et al.’s (2010) studies were particularly relevant in trying to overcome some of the limitations enumerated in Dai and Rinn’s (2008) study. They tried to understand the role of students’ perceived relative academic position in accounting for the way social comparison processes display themselves in the classroom. This aspect is greatly important for two main reasons: (1) social comparison processes underlying the BFLPE seem to be better explained by students’ perceived relative standing in their own class (Huguet et al., 2009); (2) it particularly stresses the importance of the intra-psychological elaboration of self-relevant information through perceptions and interpretations (Thijs et al., 2010). Regarding the first reason, other studies analyzed the social comparison processes underpinning the BFLPE through the meVclass ratings originally proposed by Huguet et al. (2009). These ratings were “designed to operationalize pure measures of social comparison” (Marsh et al., 2014a, p. 62), and were designed to measure how students compare to other students in the same class (Marsh et al., 2014b). Marsh et al. (2014a,b) provided support for the local dominance effect, originally studied by Zell and Alicke (2009) and Alicke et al. (2010) through the experimental manipulation of frames of reference in social comparisons. Zell and Alicke (2009) and Alicke et al. (2010) showed that people tend to choose local comparison standards over general comparison standards when comparing their performances with other people’s performances. Similarly, in the BFLPE, class-average achievement is more locally dominant than school-average achievement, and students seem to be particularly influenced by comparisons with students in their own class (Marsh et al., 2014b; Marsh and Seaton, 2015; also see Wang, 2015; Wang and Bergin, 2017 for cross-cultural analyses about the importance of students’ perceived relative standing). Regarding the second reason, the most relevant element of Thijs et al.’s (2010) model lies in explicitly addressing the role of perceptions—that is, perceived relative academic position—in mediating the impact of both classmates’ average achievement and individual achievement on academic self-concept. This, as better explained later, has been the starting point of our own investigation.

### Achievement Goal Theory

One of our main aims was to implement the existing knowledge about the BFLPE by studying the role of potentially involved dimensions drawn upon stout theoretical frameworks. Hence, Achievement Goal Theory (AGT) lent itself well to this purpose (Seaton, 2007; Wouters et al., 2015). Human beings’ striving for competence and achievement has been the main focus of several theories and studies since achievement motivation is a “ubiquitous feature of daily life” (Elliot and Church, 1997, p. 218) and one of the most important factors involved in school life and success (OECD, 2013, 2017, 2018).

Achievement motivation goals comprise beliefs, standards, and criteria related to effort, ability, and success that influence how individuals approach achievement-related situations and respond to them (Ames, 1992; Pintrich, 2000b; Elliot et al., 2017; Urdan and Kaplan, 2020). The first theorizations about the AGT conceptualized two main different types of achievement goals, traditionally conceived as contrasting (Nicholls, 1984; Dweck, 1986; Ames and Archer, 1988; Ames, 1992): mastery goals (or “task goals”), and performance goals (or “ability goals”). While mastery goals make people value learning, acquire task proficiency through effort, and strive for the development of competence, performance goals account for people’s focus on given ability, on being judged able by others, and on demonstrating competence in relation to others while outperforming them (Ames and Archer, 1988; Ames, 1992). As Ames and Archer (1988) stated, these orientations presumably differ on the basis of contextual and situational demands, which can make certain goals more salient than others. For instance, when situations make social comparison salient, students tend to focus more on levels of ability and less on effort and task strategies, which are instead the focus when absolute standards or self-improvement are emphasized (Ames and Archer, 1988; also see Senko et al., 2013; Van Yperen et al., 2015).

In the late 90s, a trichotomous variant of the achievement goal framework was formally introduced (Elliot and Harackiewicz, 1996; Elliot and Church, 1997; Middleton and Midgley, 1997; Skaalvik, 1997; Elliot, 1999; Elliot and Covington, 2001). It incorporated approach and avoidance components within performance goals. The new achievement motivation model was therefore composed of mastery goals, performance-approach goals, and performance-avoidance goals. The distinction of approach versus avoidance orientation is particularly useful when trying to understand how different achievement goals affect motivational processes (Elliot and Harackiewicz, 1994, 1996; Elliot and Church, 1997). Studies based on the dichotomous “mastery versus performance” view were less exhaustive in assessing the impact of these dimensions on motivation, indicating orientation to mastery as the only type of orientation that guarantees positive outcomes. Contrariwise, both mastery and performance-approach goals can facilitate task engagement and encourage intrinsic motivation (Elliot and Harackiewicz, 1994, 1996) and performance-approach goals can enhance graded performance, while performance-avoidance goals undermine both intrinsic motivation and graded performance (Elliot and Church, 1997; Mouratidis et al., 2018). These pivotal studies deepened our knowledge about performance-approach goals, highlighting how this dimension seems to be quite complex. Performance-approach goals, in fact, could generally be considered as a channel through which both an approach regulation and fear of failure flow. Whether the former or the latter are activated depends on the type of achievement situation (e.g., a challenging situation with little chance of failure versus a situation that presents a little chance of success) an individual happens to be in (for ulterior analyses on the complexity of performance-approach goals (see Harackiewicz et al. (1998), Hidi and Harackiewicz (2000), Pintrich (2000c), Midgley et al. (2001), Harackiewicz et al. (2002); Darnon et al. (2007), Dompnier et al. (2013); Senko and Tropiano (2016), Senko and Dawson (2017), and Senko (2019); see Pintrich (2000b) and Bardach et al. (2020), for a review on achievement motivation goals. See Scherrer et al. (2020), for a review on the development of achievement goals and their relation to academic interest and achievement during adolescence).

Shortly after, a 2 × 2 framework of achievement goals was theorized (Elliot and McGregor, 2001). Some relevant studies tried to identify an avoidance orientation in mastery goals as well, and to understand how it could differ from mastery-approach goals (e.g., Pintrich, 2000a,b; Elliot and McGregor, 2001; Van Yperen et al., 2009; Baranik et al., 2010; Senko and Freund, 2015). In fact, in certain contexts and situations, some students might be highly preoccupied with avoiding not reaching their own standards of task mastering and task understanding. This fear could result in facing academic tasks and demands in ways that are fundamentally different from those of students more oriented to mastery-approach goals (Pintrich, 2000a).

Therefore, achievement motivation can be considered as a multidimensional construct composed of different orientations, each one with different antecedents and consequences that can, in turn, account for different attitudes related to competence, achievement, and learning. Given that achievement goals are not considered as personality traits and are viewed as cognitive representations sensitive to socio-contextual aspects and demands (Pintrich, 2000b; Darnon et al., 2012; Bardach et al., 2020; Urdan and Kaplan, 2020), this uncertainty stresses the need to understand why, in each different case, mastery-approach, mastery-avoidance, performance-approach, and performance-avoidance goals show certain patterns of relations and, ultimately, why/how each one of them might have a peculiar influence on certain outcomes.

### Achievement Goals in the Big-Fish-Little-Pond Effect

In recent years, the role of achievement goals in moderating the BFLPE has been tested. For instance, aside from Wouters et al.’s (2015) study, Marsh et al. (2021) recently tested whether a set of motivational variables (e.g., achievement goals, learning strategies, and task value) could moderate the BFLPE or not. Despite showing similarities, our study and Marsh and colleagues’ study show some differences that need to be addressed. Firstly, in the present investigation, we tested the BFLPE through Thijs et al.’s (2010) model, which posits that the influence of classmates’ average achievement is better explained through the mediation of perceived relative position. Thus, we hypothesized that achievement goals would moderate the relationship between the mediator and the criterion. Because of this rationale, the present investigation differs substantially from Marsh et al.’s (2021) study. Moreover, Marsh and colleagues chose to considerate as “substantial moderators” only those variables that “would neutralize or change the direction of the BFLPE” (p. 18), and claimed that they considered the BFLPE to remain consistent if the size of the moderation was “less than half the size of the BFLPE” (Marsh et al., 2021, p. 3). Because this was an arbitrary operationalization, it is not the same rationale we used to interpret our results. Finally, Marsh et al. (2021) also stated that “neither our study nor any finite set of studies can prove that there are no student-level moderators of the BFLPE” (p. 17), pointing out that there are other variables, not considered as potential moderators in their study, that could moderate the effect. We believe that this statement could also be true for the moderators already considered in Marsh et al.’s (2021; e.g., achievement motivation variables) study. Contextual and situational differences, as well as differences in samples, could render certain intra-psychological moderators more relevant in influencing the BFLPE. Assuming that certain variables certainly do (or do not) moderate the effect based on results of finite sets of studies might be misleading.

### The Present Study

Therefore, the present study drew upon Thijs et al.’s (2010) model, in which the effect of classmates’ average achievement and individual achievement on perceptions about oneself was mediated by the perceived relative position in the classroom. Moreover, we tried to deepen our understanding of social comparison processes by assessing the impact of achievement goals on how perceived relative position influences perceptions about possibly having a better future than classmates. The classroom was chosen as the privileged focus to test the BFLPE because of the salience that it holds in students’ social comparison processes, which seem to be better explained by taking into account the students’ perceived relative standing in their own class (Huguet et al., 2009; Thijs et al., 2010; Wang, 2015; Wang and Bergin, 2017). Perceptions about the future were taken into account as the criterion variable because of the importance that present social comparisons hold for dimensions related to this aspect of self-perception as well (e.g., Davis, 1966; Marsh and Yeung, 1997; Parker et al., 2014). Moreover, we chose to test the impact of the BFLPE on future expectations in order to test its generalizability to constructs other than academic self-concept, and analyze its potential negative effect on something as important as students’ expectations (OECD, 2013). In particular, we focused on the perception of possibly having a better future than classmates.

We hypothesized that perceived relative position would mediate the impact of classmates’ average achievement and individual average achievement on the perception of possibly having a better future than classmates. As explained above, previous studies (e.g., Huguet et al., 2009; Thijs et al., 2010; Marsh et al., 2014b; Wang, 2015; Wang and Bergin, 2017) showed that perceived relative position actually mediates the effects of classmates’ average achievement and individual average achievement in the BFLPE, and renders the active process through which students evaluate their classmates’ achievements, and their own achievement, explicit (Thijs et al., 2010). Hence, our rationale was that students would recur to both their grades and their classmates’ average grades to assess their relative position in the classroom. In particular, we expected that classmates’ average achievement would have a negative effect on perceived relative position, whereas individual achievement would have a positive impact on it. We also expected that a high perceived relative position would be positively related to perceptions about the future (hypothesis 1).

Then, differing from Wouters et al.’s (2015) study, we hypothesized that each type of goal could have a specific role in the social comparison process at issue. In particular, we hypothesized that at high levels of performance-approach goals, the effect of unflattering social comparison—explained through a low perceived relative position—on the criterion would be worse than the effect at low levels of performance-approach goals (hypothesis 2). This effect was hypothesized drawing on findings that showed how these goals can be linked to outcomes favorable for the self (e.g., Elliot and Harackiewicz, 1996; Elliot and Church, 1997; Barron and Harackiewicz, 2001, 2003; Harackiewicz et al., 2002).

We also hypothesized that at high levels of performance-avoidance goals, the effect of unflattering social comparison (low perceived relative position) on the criterion would be worse than the effect at low levels of performance-avoidance goals (hypothesis 3). As literature about AGT generally suggests (e.g., Elliot and Harackiewicz, 1996; Elliot and Church, 1997; Elliot, 1999), we in fact expected performance-avoidance goals to result in a maladaptive outcome.

**FIGURE 1:**
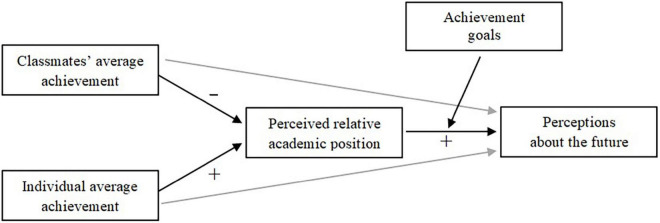
Research design.

Finally, we hypothesized that mastery-approach goals (hypothesis 4) and mastery-avoidance goals (hypothesis 5) would not have a role in the social comparison process at issue. Even if some studies found that holding mastery goals can also be linked to social comparisons (e.g., Régner et al., 2007; Darnon et al., 2010; Wouters et al., 2015), this dimension is mostly related to motivation toward learning and self-improvement *per se*, without being necessarily involved in aspects like wanting to demonstrate said ability, or wanting to be better than others (Ames and Archer, 1988; Ames, 1992), which are dynamics more involved in social comparison processes. The research design is shown in Figure 1.

## Materials and Methods

### Procedure and Participants

For the present investigation, many high schools were approached over the years 2016–2018. The schools who agreed to participate were provided with the following: an access request, in which the characteristics of the study were described and the use of the data for solely research purposes was ensured; a presentation of the BFLPE and our ongoing research findings; the questionnaire. Nine classical, linguistic, and scientific lyceums agreed to participate. The final sample was composed of students from different regions of Italy, attending relatively differentiated types of high schools, for a sample of 689 high school students (see Appendix B for ulterior information about the number of classrooms and students per class). Students were presented with the questionnaire and they completed it in their respective classrooms. All the students were given one hour to fill in the questionnaire. Since the participants were all students already of age, they gave their consent before filling in the questionnaire and participation was voluntary. During the administration of the questionnaire, teachers were asked to leave the classrooms not to influence the students’ answers with their presence, and only one or two researchers remained with the students.

The only personal information students were asked to provide was their age and their gender. With regard to the first feature, 683 out of 689 students specified their ages (*M*_age_ = 18.15; *SD* = 0.57). The highest percentage of the sample consisted of 18-year-olds (*n* = 485; 71.0%), while the lowest percentage consisted of 21-year-olds (*n* = 2; 0.2%). Concerning gender, 680 of the 689 students (98.69%) specified their gender. The percentages of male students (*n* = 355; 52.2%) and female students (*n* = 325; 47.8%) were similar. Therefore, the sample was fairly balanced with regard to gender differences.

### Measures

The questionnaire was structured as follows.

#### Individual Achievement and Classmates’ Achievement

Individual academic achievement and classmates’ average achievement were self-reported by students through two items: “What was your grade point average at the end of the previous year?” and “What was your classmates’ overall grade point average at the end of the previous year?” Because we could not access this information through official academic records, each student had the opportunity to indicate their own average achievement and their classmates’ average achievement. Thus, we gave importance to the knowledge and the perception that each student had about these aspects. Because Italian upper secondary schools use a 10-point grading scale to assess students’ performances in each subject, students had to indicate their average achievement and their classmates’ average achievement through a 10-point scale.

#### Perceived Relative Position

To make the comparison process explicit (Dai and Rinn, 2008; Thijs et al., 2010), one item explicitly referred to a student’s school ability compared to that of her/his other classmates. The item ‘‘To what degree do you think you are better than your classmates?’’^1^ was designed in order to allow each student to actually consider her/his relative academic position. Students had to answer it using a 10-point Likert scale.

#### Achievement Goals

The 12-item scale used to assess achievement motives/goals was derived and adapted from Elliot and McGregor’s (2001) measure through a systematic translation of the items (see also Darnon and Butera, 2005; Wang et al., 2007). The items were translated from English to Italian by a bilingual professor and back-translated by another bilingual professor. The two English versions were then compared to resolve discrepancies. The final version of the scale was translated into Italian by both professors. The two major dimensions of achievement motivation that the scale examines are mastery goals and performance goals. Furthermore, for each dimension, both approach and avoidance aspects can be noticed. Thus, the scale allows observing individuals’ orientations toward four types of goals: mastery-approach, mastery-avoidance, performance-approach, and performance-avoidance. Students had to answer using a 5-point Likert scale, according to their agreement or disagreement with each item.

**FIGURE 2:**
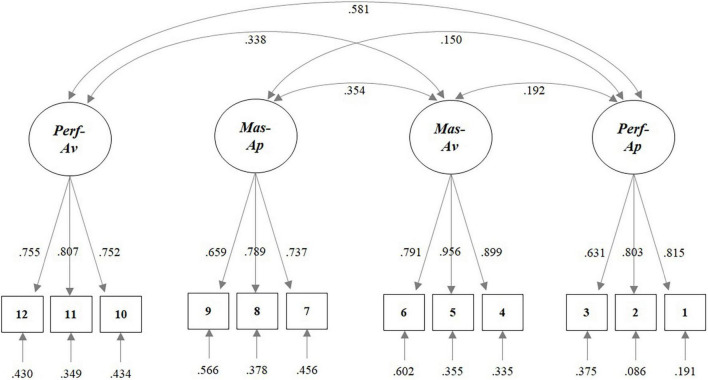
Factorial structure of the Achievement Goals scale.

In order to confirm the factorial structure of the scale (*n* = 684), we ran a CFA with Mplus 8 (Muthén and Muthén, 2017) using robust maximum likelihood estimation (see Figure 2). To assess the goodness of fit, we considered the following indices: chi-square statistic (χ^2^), root mean squared error of approximation (RMSEA) and 90% RMSEA confidence interval (CI), Comparative Fit Index (CFI), Tucker–Lewis Index (TLI), and standardized root-mean squared residual (SRMR). We selected the cut-off values for each index following Schreiber et al.’s guidelines (Schreiber et al., 2006): Chi-*P* ≥ 0.05; RMSEA < 0.06–0.08; CFI ≥ 0.95; TLI ≥ 0.95; SRMR ≤ 0.08. The results of the final four-factor CFA model (see Appendix A for scale items) showed a good fit of the model to the data: χ^2^ = 155.26, *p* < 0.001, df = 48; RMSEA = 0.06, 90% CI = [0.05, 0.07]; CFI = 0.96; TLI = 0.95; SRMR = 0.05. Standardized factor loadings ranged from 0.63 to 0.96 (factor loadings are reported in Appendix A). Latent factor correlations were all significant and positive, except for the correlation between mastery-approach and performance-avoidance goals (*r* = 0.02, *p* = 0.65). Performance-approach and performance-avoidance goals correlated strongly (*r* = 0.58, *p* < 0.001). Mastery-approach and mastery-avoidance goals showed a moderate correlation (*r* = 0.35, *p* < 0.001), and the same went for performance-avoidance and mastery-avoidance goals (*r* = 0.34, *p* < 0.001). Weaker correlations were found between mastery-avoidance and performance-approach goals (*r* = 0.19, *p* < 0.001) and between mastery-approach and performance-approach goals (*r* = 0.15, *p* = 0.001). Reliability was tested and each dimension showed acceptable to good levels of reliability: α_*mastery–approach*_ = 0.77; α*_*mastery–avoidance*_* = 0.79; α_*performance–approach*_ = 0.91; and α_*performance–avoidance*_ = 0.81.

### Future Expectations

The specific aspect of perceptions about the future at issue is the degree to which each student perceived s/he was potentially going to have a better future than her/his classmates. It was noted through the item “To what degree do you think you are going to have a better future than your classmates?,” which is tightly related to the social comparison dynamics that might emerge in a specific class group. Students had to answer using a 10-point Likert scale. In the following, we will refer to this variable using both “better future than classmates” and the shortened term “better future.”

The data collected were analyzed using SPSS Statistics – 25 (IBM SPSS, 2017). Hayes’s PROCESS macro was used to test all of the hypotheses and, in particular, PROCESS Model 4 (Hayes, 2013) was used to assess our first hypothesis, while PROCESS Model 14 (Hayes, 2013) was used to assess the other four hypotheses. Conditional process modeling was therefore used to explore moderated mediation, in which the effects of classmates’ average achievement and individual achievement (predictors) on perceptions about possibly having a better future than classmates (criterion) via perceived relative position (M) is moderated by different aspects of achievement goals (W).

## Results

### Preliminary Analyses

Before proceeding with analyses regarding moderated mediations, all the variables considered in the study were standardized and correlations among them were assessed. They are shown below in Table 1.

**TABLE 1:** Descriptive statistics and bivariate correlations.

	*M*	*SD*	1.	2.	3.	4.	5.	6.	7.	8.
**1**. Classmate’s achievement	7.08	0.74	–							
**2**. Individual achievement	7.38	0.95	0.18**	–						
**3**. Relative position	5.54	2.38	−0.13**	0.34**	–					
**4**. Performance- approach	1.17	1.04	−0.10*	0.13**	0.38**	–				
**5**. Performance-avoidance	1.44	1.12	−0.11**	−0.09*	0.14**	0.53**	–			
**6**. Mastery-approach	3.08	0.78	0.01	0.20**	0.06	0.13**	0.02	–		
**7.** Mastery-avoidance	2.32	0.95	–0.06	−0.10**	−0.13**	0.16**	0.26**	0.32**	–	
**8**. Better future	5.86	2.17	−0.09*	0.12**	0.67**	0.32**	0.22**	–0.04	–0.07	–

*p < 0.05; **p < 0.01.

As expected, perceived relative position negatively correlated with classmates’ average achievement (*r* = −0.13; *p* < 0.001) and positively correlated with individual achievement (*r* = 0.34; *p* < 0.001). Moreover, perceived relative position was significantly and positively correlated with better future (*r* = 0.67; *p* < 0.001). Concerning the different aspects of achievement goals, we found that performance-approach and performance-avoidance goals showed a positive correlation (*r* = 0.53; *p* < 0.001), and the same went for mastery-approach and mastery-avoidance goals (*r* = 0.32; *p* < 0.001). Interestingly, perceptions about possibly having a better future than classmates showed a significant and positive correlation with both performance-approach goals (*r* = 0.32; *p* < 0.001) and performance-avoidance goals (*r* = 0.21; *p* < 0.001), while its correlation with mastery-approach and mastery-avoidance goals was not significant. Perceived relative position showed a moderate correlation with performance-approach goals (*r* = 0.38; *p* < 0.01).

### Simple Mediation

PROCESS Model 4 (Hayes, 2013) was run to assess whether perceived relative position mediated the relationship between the predictors and the criterion. We first considered classmates’ average achievement as the predictor and individual achievement as the covariate. Both the predictors significantly impacted perceived relative position and the overall model was significant [*F*_(2,658)_ = 58.51; *R*^2^ = 0.15; *p* < 0.001]. Consistent with Thijs et al.’s (2010) model, the effect of classmates’ average achievement on perceived relative position was negative [Path a_class_: *B* = −0.20; *SE* = 0.04; 95% CI (−0.27, −0.13); *p* < 0.001], whereas individual achievement’s effect was positive [Path a_ind_: *B* = 0.37; *SE* = 0.04; 95% CI (0.30, 0.45); *p* < 0.001]. Concerning the effect of perceived relative position on the criterion, the effect was significant and positive [Path b: *B* = 0.71; *SE* = 0.03; 95% CI (0.65, 0.77); *p* < 0.001]. The indirect effect was significant [*B* = −0.14; *SE* = 0.02; 95% CI (−0.19, −0.10)]. We then tested the same model considering individual achievement as the predictor and classmates’ average achievement as the covariate, and the indirect effect was significant in this case as well [*B* = 0.27; *SE* = 0.03; 95% CI (0.21, 0.33)]. Hence, perceived relative position was confirmed to mediate the impact of classmates’ average achievement and individual achievement on the perception of possibly having a better future than classmates. Our first hypothesis was therefore confirmed.

### Moderated Mediation: Performance-Approach Goals

To assess our second hypothesis, PROCESS Model 14 (Hayes, 2013) was run, introducing performance-approach goals as the moderator of the effect of perceived relative position on better future than classmates (Path b).

In the first analysis run, classmates’ average achievement was introduced in the model as the predictor, while individual achievement was introduced as the covariate. The overall model was significant [*F*_(5,654)_ = 115.55; *R*^2^ = 0.47; *p* < 0.001]. Perceived relative position significantly mediated the impact of classmates’ average achievement and individual achievement on the perception of possibly having a better future than classmates [Path b: *B* = 0.67; *SE* = 0.03; 95% CI (0.60, 0.73); *p* < 0.001]. For what concerns performance-approach goals, its direct effect on the criterion was significant and positive [*B* = 0.11; *SE* = 0.03; 95% CI (0.05, 0.17); *p* = 0.001]. Most importantly, the interaction between the mediator and the moderator (relative perceived position*performance-approach goals) resulted significantly associated with the dependent variable [*B* = −0.07; *SE* = 0.03; 95% CI (−0.12, −0.01); *p* = 0.045].

The index of moderated mediation, which shows if a certain “moderator variable has a non-zero weight in the function linking the indirect effect of X on Y through M to the moderator” (Hayes, 2015, p. 3), was significant [index = 0.01; *SE* = 0.01; 95% CI (0.00, 0.03)], upholding moderated mediation and showing that the differences at high versus low values of the moderator were significant. In particular, at +1 *SD* of performance-approach goals, the conditional indirect effect was significant and negative [*B* = −0.12; bootstrapped *SE* = 0.02; 95% CI (−0.17, −0.07)], and the same went for the indirect effect at −1 *SD* of the moderator [*B* = −0.15; bootstrapped *SE* = 0.03; 95% CI (−0.20, −0.10)], but there was a larger effect at low levels of performance-approach goals.

We then ran the same analysis introducing individual achievement as the predictor and classmates’ average achievement as the covariate. For what concerns the index of moderated mediation, it was significant [index = −0.03; *SE* = 0.01; 95% CI (−0.05, −0.00)] confirming, in this case as well, moderated mediation (Hayes, 2015) and showing that the differences at high versus low values of the moderator were significant. In particular, at +1 *SD* of performance-approach goals, the indirect effect was significant and positive [*B* = 0.23; bootstrapped *SE* = 0.04; 95% CI (0.16, 0.30)], and the same went for the indirect effect at −1 *SD* of the moderator [*B* = 0.28; bootstrapped *SE* = 0.03; 95% CI (0.22, 0.34)]. The effect was larger at low levels of performance-approach goals.

Through a simple slope analysis and the Johnson-Neyman method, we were able to elaborate on the conditional effect of perceived relative academic position at different values of performance-approach goals.

**FIGURE 3:**
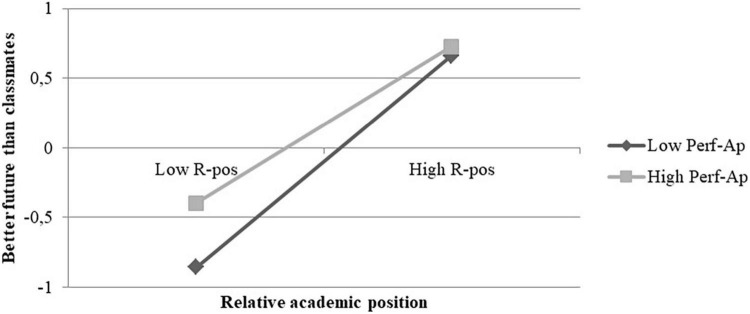
Conditional effects of perceived relative academic position (R-Pos) on better future at different levels of performance-approach (Perf-Ap).

The Johnson-Neyman analysis showed that there were no significant transition points within the observed range of the moderator, therefore all levels of performance-approach goals moderated the effect (see Appendix C). Conditional effects showed that the effect was larger at low levels of performance-approach goals [*B* = 0.73; 95% CI (0.65, 0.81); *p* < 0.001] than at high levels of the moderator [*B* = 0.60; 95% CI (0.50, 0.70); *p* < 0.001]. Moreover, the slopes shown in Figure 3 suggested that students who are scarcely oriented to performance-approach particularly might suffer from a low perceived relative position. In light of the above, our third hypothesis can be considered confirmed.

### Moderated Mediation: Performance-Avoidance Goals

The role of performance-avoidance goals was then examined through PROCESS Model 14 (Hayes, 2013), introducing this variable as the moderator of the path between perceived relative position and better future than classmates (Path b).

In the first analysis run, classmates’ average achievement was introduced in the model as the predictor, while individual achievement was introduced as the covariate. The overall model was significant [*F*_(5,654)_ = 120.88; *R*^2^ = 0.48; *p* < 0.001] and, in this case as well, perceived relative position mediated the impact of classmates’ achievement and individual achievement on the criterion [Path b: *B* = 0.68; *SE* = 0.03; 95% CI (0.62, 0.74); *p* < 0.001]. Just like performance-approach goals, the direct impact of performance-avoidance goals on the criterion was significant and positive [*B* = 0.13; *SE* = 0.03; 95% CI (0.08, 0.19); *p* < 0.001]. Most importantly, the interaction between this dimension and the mediator (relative perceived position) was significant [*B* = −0.09; *SE* = 0.03; 95% CI (−0.14, −0.03); *p* < 0.01].

The index of moderated mediation showed that the indirect effect in question was a linear function of the moderator (Hayes, 2015), meaning that moderated mediation was upheld and the differences of the effect at high versus low values of performance-avoidance goals were significant [index = 0.02; *SE* = 0.02; 95% CI (0.00, 0.03)]. The differences in the magnitude of the conditional indirect effects at high (+1 *SD*) versus low (−1 *SD*) levels of the moderator were examined. At +1 *SD* of performance-avoidance goals, the effect was significant and negative [*B* = −0.12; bootstrapped *SE* = 0.02; 95% CI (−0.17, −0.08)], and the same went for the indirect effect at −1 *SD* of the moderator [*B* = −0.15; bootstrapped *SE* = 0.03; 95% CI (−0.21, −0.10)]. The effect was larger at low levels of performance-avoidance goals.

We then ran the same analysis introducing individual achievement as the predictor and classmates’ average achievement as the covariate. The index of moderated mediation was significant [index = −0.03; *SE* = 0.01; 95% CI (−0.06, −0.01)], confirming moderated mediation and showing that the differences of the effect at high versus low values of performance-avoidance goals were significant. At +1 *SD* of the moderator, the effect was significant and positive [*B* = 0.22; bootstrapped *SE* = 0.03; 95% CI (0.16, 0.29)]. At −1 *SD* of the moderator, the effect was significant and positive as well [*B* = 0.29; bootstrapped *SE* = 0.03; 95% CI (0.22, 0.36)], and larger than the effect at higher values of the moderator.

Through a simple slope analysis and the Johnson-Neyman method, we further examined the impact of perceived relative academic position on perceptions about the future at different levels of performance-avoidance goals.

**FIGURE 4:**
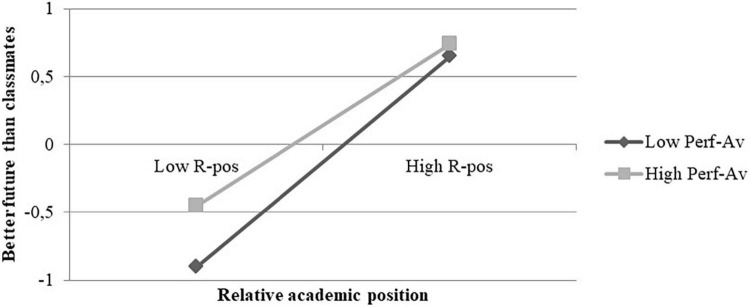
Conditional effects of perceived relative academic position (R-Pos) on better future at different levels of performance-avoidance (Perf-Av).

The Johnson-Neyman analysis showed that there were no significant transition points within the observed range of the moderator. Therefore, all levels of performance-avoidance moderated the effect (see Appendix D). Conditional effects showed that at low levels of the moderator the effect was larger [*B* = 0.77; 95% CI (0.69, 0.84); *p* < 0.001] than at high levels of the moderator [*B* = 0.59; 95% CI (0.50, 0.68); *p* < 0.001]. Moreover, as shown in Figure 4, the impact of performance-avoidance goals in moderating the effect was similar to the impact of performance-approach goals. In fact, students who perceived they were academically worse than their classmates (low perceived position) had worse future expectations when they were scarcely oriented to performance-avoidance goals. Therefore, these results do not confirm our third hypothesis.

### Moderated Mediation: Mastery-Approach Goals

To assess our fourth hypothesis, PROCESS Model 14 (Hayes, 2013) was run, introducing mastery-approach goals as the moderator of the path between perceived relative position and better future (Path b). Mastery-approach goals did not have a significant direct effect on the criterion [*B* = −0.06; *SE* = 0.03; *p* = 0.06]. Most importantly, the interaction between the mediator and the moderator was not significant [*B* = 0.05; *SE* = 0.03; *p* = 0.08]. The index of moderated mediation was non-significant when considering classmates’ achievement as the main predictor [index = −0.01; *SE* = 0.01; 95% CI (−0.02, 0.00)] and when considering individual achievement as the main predictor as well [index = 0.02; *SE* = 0.01; 95% CI (−0.00, 0.04)]. This result shows, as expected, that the indirect effect is not contingent on mastery-approach goals. Therefore, these goals do not have a role in the social comparison process at issue, being a dimension that is more involved in aspects related to learning and self-improvement *per se*. Our fourth hypothesis was therefore confirmed.

### Moderated Mediation: Mastery-Avoidance Goals

To assess our fifth hypothesis, PROCESS Model 14 (Hayes, 2013) was run, introducing mastery-avoidance goals as the moderator of Path b. Similarly to results concerning mastery-approach goals, mastery-avoidance goals did not have a significant direct effect on the criterion (*B* = 0.02; *SE* = 0.03; *p* = 0.43). Most importantly, there was no moderation (*B* = −0.02; *SE* = 0.03; *p* = 0.55). The index of moderated mediation was non-significant when considering classmates’ achievement as the main predictor [index = 0.00; *SE* = 0.01; 95% CI (−0.01, 0.02)] and when considering individual achievement as the main predictor as well [index = −0.01; *SE* = 0.01; 95% CI (−0.03, 0.02)]. These results show that mastery-avoidance goals, just like mastery-approach goals, do not have a role in the social comparison process at issue. Our fifth hypothesis was therefore confirmed.^2^

## Discussion

Our study’s main aim was to replicate Thijs et al.’s (2010) model with a sample of Italian students to test the presence of the BFLPE in our educational system. We also sought to test whether perceived relative position mediated the relationship between individual and classmates’ average achievement and the perception about possibly having a better future than classmates. The results shown in the previous section confirmed that both classmates’ achievement and individual achievement impacted perceived relative position. While the first dimension negatively impacted it, the latter had a positive effect on it. In turn, perceived relative position in the classroom had a significant, positive effect on the aspect of perceptions about the future at issue. Therefore, in the self-evaluation processes that take place in classrooms, students actively evaluate how they perform compared to their classmates. This active evaluation must be considered when studying social comparison processes in school settings. Analyzing students’ perceptions and active elaboration of information might be more revealing than studying how schoolmates’ performance alone directly influences the self and self-related outcomes.

Our second aim was to understand whether different individual motivational orientations—mastery-approach, mastery-avoidance, performance-approach, and performance-avoidance goals—could each have a specific role in the social comparison process analyzed. The findings presented above support the idea that performance-approach goals might also result in adaptive outcomes, as advocated by the multiple goal perspective. The findings also imply that performance-approach goals and mastery goals might simply have a different impact on different types of outcomes and among students with certain socio-psychological characteristics (Harackiewicz et al., 2000; Barron and Harackiewicz, 2001, 2003; Harackiewicz et al., 2002; Darnon et al., 2018; Mouratidis et al., 2018). Generally speaking, the effects that different kinds of goals result in are likely to depend on the contextual characteristics in which said goals are pursued and on these contexts’ incidental requests. Moreover, these results supported the findings of previous works (e.g., Harackiewicz et al., 1997, 1998) that showed how, for students attending secondary and tertiary education, pursuing performance-approach goals might positively affect diverse outcomes.

Mastery goals did not moderate the effect of perceived relative position on future expectations, and this result confirmed that the relationships among performance-approach goals, mastery goals, and school-related outcomes are more complex and dynamic than what was firstly theorized in the normative goal theory (Barron and Harackiewicz, 2001, 2003). It seems that, at the particular point in time in which the data have been collected (i.e., at the end of the last year of high school), performance-approach goals are more relevant than mastery goals when students evaluate certain aspects of their own future possibilities. This also means that pursuing certain types of goal orientations might be more appropriate in situations where particular requests are more salient than others (Zimmerman and Kitsantas, 1999; Barron and Harackiewicz, 2003).

An interesting—yet perhaps unsettling—result is the one regarding performance-avoidance goals. In fact, it emerged that, just like performance-approach goals, in the specific process of social comparison analyzed, trying to avoid failure and being considered less achieving than peers might actually help students feel like they can have a better future than their classmates, even when their present relative academic position is not that high. Perhaps, at the end of secondary education, when students are about to get the final grade that will project them toward tertiary education or the labor market, pursuing performance-avoidance goals alongside performance-approach goals might help them feel more secure about outperforming their class peers after school. This aspect surely needs further examination, especially given that performance-avoidance goals are usually related to negative outcomes.

The present investigation has some limitations that have to be considered. Firstly, the data were collected at a single point in time and are therefore cross-sectional, making it impossible to make assumptions about causality. Despite using cross-sectional designs can be useful and helpful in several areas of inquiry (Spector, 2019), numerous studies highlighted the importance of recurring to longitudinal data when testing mediation (e.g., Maxwell et al., 2011; Jose, 2016). It would be interesting—if not necessary—to consider data over lower grades up to higher grades to assess how the dimensions considered in the study change over time and if causal relationships among them exist.

Another limitation concerns the fact that both individual achievement and classmates’ average achievement were self-reported by students. Even though numerous studies showed that students’ estimations and self-reports of diverse types of data are actually reliable (e.g., Crockett et al., 1987), and even if priority has been given to each student’s own perception about these dimensions, in future research it might be useful to resort to standardized tests too to take note of these data.

Moreover, the BFLPE is usually analyzed through doubly-latent multilevel models. The type of analyses carried out in the present investigation might be useful in giving an insight on the relationships among the dimensions considered, but do not allow controlling for the nesting of students within classes, nor for latent correlations among variables. When considering this study’s results, this must be taken into account.

Finally, given that the dimension of future expectations considered in the present study is very specific, it might be important to consider other aspects that social comparisons might influence. Some studies analyzed the influence that social comparison processes have on perceptions about future possibilities (e.g., Davis, 1966; Marsh and Yeung, 1997; Parker et al., 2014). However, research on the BFLPE has usually focused on the impact that these processes have on present academic self-concept and self-construals (Marsh et al., 1988; Marsh, 1990, 1993; Marsh et al., 1995; Marsh et al., 2007), and how these constructs result in numerous other important outcomes (e.g., Craven and Marsh, 2008). On the other hand, taking into account other aspects related to the self might be useful in deepening our understanding of how each motivational orientation actually holds different effects for different outcomes. For example, as said before, mastery goals might have a role when considering outcomes related to self-improvement, to the need for competence, and less linked to the dimensions of performance and outperformance.

## Conclusion

The present investigation, albeit lending itself to improvement, showed some interesting findings that should be considered to expand our knowledge about the BFLPE and social comparison processes in school settings. Firstly, it confirmed the importance of considering the role of the local frame of reference—that is, the classroom—in social comparison processes and how these social comparison processes are well accounted for by perceived relative academic position. Secondly, drawing upon literature regarding achievement motivation, it was possible to understand how—and how differently—individual goal orientations came into play in influencing the perceptions about the future at issue. These findings, apart from supporting research about how different goals have specific influences on specific outcomes in specific situations, enrich research on the BFLPE in trying to understand how school-related dimensions moderate the effect. These aspects should be considered in further research regarding both the AGT and the BFLPE.

## Data Availability Statement

The raw data supporting the conclusions of this article will be made available by the authors, without undue reservation.

## Ethics Statement

The studies involving human participants were reviewed and approved by Department of Developmental and Social Psychology, University of Rome “La Sapienza.” The patients/participants provided their written informed consent to participate in this study.

## Author Contributions

AC and SL conceptualized and designed the study. AC, FS, and MM administered the questionnaires, organized the database, and contributed to writing sections of the manuscript. AC, FS, and SL performed the statistical analysis. AC wrote the first draft of the manuscript. All authors contributed to revising the manuscript and approved the submitted version.

## Conflict of Interest

The authors declare that the research was conducted in the absence of any commercial or financial relationships that could be construed as a potential conflict of interest.

## Publisher’s Note

All claims expressed in this article are solely those of the authors and do not necessarily represent those of their affiliated organizations, or those of the publisher, the editors and the reviewers. Any product that may be evaluated in this article, or claim that may be made by its manufacturer, is not guaranteed or endorsed by the publisher.

## Footnotes

1 The English translation rather loses the nuance that the item has in Italian: the word “better” has to be interpreted, in fact, as referring to ability levels. The Italian item is: “*Quanto pensi di essere più bravo rispetto ai tuoi compagni di classe*?”

2 Despite not being the main focus of the present investigation, we also tested whether achievement goals moderated the effect of classmates’ average achievement on future expectations. For each dimension of achievement goals, we ran PROCESS model 1, which tests simple moderation, and then ran PROCESS model 15, which enables to test moderation on the path b and path c^1^ of the mediation (perceived relative position mediating the effect of individual achievement and classmates’ achievement on better future). Concerning simple moderation, the interaction of classmates’ average achievement and performance-approach goals was significant [*B* = 0.08; *SE* = 0.04; 95% CI (0.00, 0.15); *p* = 0.05]. Conditional effects showed that the moderation was significant only at low levels (−1 *SD*) of performance-approach [*B* = −0.15; bootstrapped *SE* = 0.06; 95% CI (−0.26, −0.03)]. In particular, the Johnson-Neyman output showed that the point at which the moderation transitioned from statistically significant to non-significant was performance-approach = −0.08. Above this value, conditional effects were non-significant. Moreover, the Johnson-Neyman output showed that low performance-approach goals worsened classmates’ average achievement’s negative effect on the criterion. Performance-avoidance goals did not moderate the effect of classmates’ average achievement on future expectations [*B* = 0.06; *SE* = 0.04; 95% CI (−0.01, 0.13); *p* = 0.08]. Mastery-approach [*B* = −0.02; *SE* = 0.04; 95% CI (−0.01, 0.05); *p* = 0.53] and mastery-avoidance [*B* = 0.05; *SE* = 0.04; 95% CI (−0.03, 0.12); *p* = 0.24] goals as well, as expected, did not moderate the effect of classmates’ average achievement on future expectations. We then ran PROCESS model 15. As already explained before, performance-approach goals moderated perceived position’s effect on future expectations [*B* = −0.07; *SE* = 0.03; 95% CI (−0.12, −0.01); *p* < 0.05]. Instead, they did not moderate classmates’ average achievement direct effect on the criterion [*B* = 0.04; *SE* = 0.03; 95% CI (−0.02, 0.10); *p* = 0.14]. The index of moderated mediation was significant [index = 0.01; *SE* = 0.01; 95% CI (0.00, 0.03)]. Similarly, performance-avoidance goals moderated the impact of perceived relative position on future expectations [*B* = −0.09; *SE* = 0.03; 95% CI (−0.14, −0.03); *p* < 0.01], but did not moderate the effect of classmates’ achievement on future expectations [*B* = 0.01; *SE* = 0.03; 95% CI (−0.04, 0.07); *p* = 0.14]. The index of moderated mediation was significant [index = 0.02; *SE* = 0.01; 95% CI (0.00, 0.03)]. Mastery-approach and mastery-avoidance goals did not moderate neither the model’s path b, nor its path c^1^.

## References

[B1] Alicke M. D. Zell E. Bloom D. L. (2010). Mere categorization and the frog-pond effect. *Psychol. Sci.* 21 174–177. 10.1177/0956797609357718 20424040

[B2] Ames C. (1992). Classrooms: goals, structures, and student motivation. *J. Educ. Psychol.* 84 261–271. 10.1037/0022-0663.84.3.261

[B3] Ames C. Archer J. (1988). Achievement goals in the classroom: students’ learning strategies and motivation processes. *J. Educ. Psychol.* 80 260–267. 10.1037/0022-0663.80.3.260

[B4] Baranik L. E. Stanley L. J. Bynum B. H. Lance C. E. (2010). Examining the construct validity of mastery-avoidance achievement goals: a meta-analysis. *Hum. Perform.* 23 265–282. 10.1080/08959285.2010.488463

[B5] Bardach L. Oczlon S. Pietschnig J. Lüftenegger M. (2020). Has achievement goal theory been right? A meta-analysis of the relation between goal structures and personal achievement goals. *J. Educ. Psychol.* 112 1197–1220. 10.1037/edu0000419

[B6] Barron K. E. Harackiewicz J. M. (2001). Achievement goals and optimal motivation: testing multiple goal models. *J. Pers. Soc. Psychol.* 80 706–722. 10.1037/0022-3514.80.5.706 11374744

[B7] Barron K. E. Harackiewicz J. M. (2003). Revisiting the benefits of performance-approach goals in the college classroom: exploring the role of goals in advanced college courses. *Int. J. Educ. Res.* 39 357–374. 10.1016/j.ijer.2004.06.004

[B8] Craven R. G. Marsh H. W. (2008). The centrality of the self-concept construct for psychological wellbeing and unlocking human potential: implications for child and educational psychologists. *Educ. Child Psychol.* 25 104–118.

[B9] Crockett L. J. Schulenberg J. E. Petersen A. C. (1987). Congruence between objective and self-report data in a sample of young adolescents. *J. Adolesc. Res.* 2 383–392. 10.1177/074355488724006

[B10] Dai D. Y. Rinn A. N. (2008). The big-fish-little-pond effect: What do we know and where do we go from here? *Educ. Psychol. Rev.* 20 283–317. 10.1007/s10648-008-9071-x

[B11] Darnon B. Butera F. (2005). Buts d’accomplissement, stratégies d’étude, et motivation intrinsèque: présentation d’un domaine de recherche et validation française de l’échelle d’Elliot et McGregor (2001). *L’année Psychol.* 105 105–131. 10.3406/psy.2005.3821

[B12] Darnon C. Dompnier B. Gilliéron O. Butera F. (2010). The interplay of mastery and performance goals in social comparison: a multiple-goal perspective. *J. Educ. Psychol.* 102 212–222. 10.1037/a0018161

[B13] Darnon C. Dompnier B. Marijn Poortvliet P. (2012). Achievement goals in educational contexts: a social psychology perspective. *Soc. Pers. Psychol. Compass* 6 760–771. 10.1111/j.1751-9004.2012.00457.x

[B14] Darnon C. Harackiewicz J. M. Butera F. Mugny G. Quiamzade A. (2007). Performance-approach and performance-avoidance goals: When uncertainty makes a difference. *Pers. Soc. Psychol. Bull.* 33 813–827. 10.1177/0146167207301022 17488870

[B15] Darnon C. Jury M. Aelenei C. (2018). Who benefits from mastery-approach and performance-approach goals in college? Students’ social class as a moderator of the link between goals and grade. *Eur. J. Psychol. Educ.* 33 713–726. 10.1007/s10212-017-0351-z

[B16] Davis J. A. (1966). The campus as a frog pond: an application of theory of relative deprivation to career decisions for college men. *Am. J. Soc.* 72 17–31. 10.1086/224257

[B17] Dompnier B. Darnon C. Butera F. (2013). When performance-approach goals predict academic achievement and when they do not: a social value approach. *Br. J. Soc. Psychol.* 52 587–596. 10.1111/bjso.12025 23336439

[B18] Dweck C. S. (1986). Motivational processes affecting learning. *Am. Psychol.* 41 1040–1048. 10.1037/0003-066X.41.10.1040

[B19] Elliot A. J. (1999). Approach and avoidance motivation and achievement goals. *Educ. Psychol.* 34 169–189. 10.1207/s15326985ep3403_3

[B20] Elliot A. J. Church M. A. (1997). A hierarchical model of approach and avoidance achievement motivation. *J. Pers. Soc. Psychol.* 72 218–232. 10.1037/0022-3514.72.1.218 10234849

[B21] Elliot A. J. Covington M. V. (2001). Approach and avoidance motivation. *Educ. Psychol. Rev.* 13 73–92. 10.1023/A:1009009018235

[B22] Elliot A. J. Dweck C. S. Yeager D. S. (2017). *Handbook of Competence and Motivation: Theory and Application*, 2nd Edn. New York, NY: The Guilford Press.

[B23] Elliot A. J. Harackiewicz J. M. (1994). Goal setting, achievement orientation, and intrinsic motivation: a mediational analysis. *J. Pers. Soc. Psychol.* 66 968–980. 10.1037/0022-3514.66.5.968 8014838

[B24] Elliot A. J. Harackiewicz J. M. (1996). Approach and avoidance achievement goals and intrinsic motivation: a mediational analysis. *J. Pers. Soc. Psychol.* 70 461–475. 10.1037/0022-3514.70.3.461 8014838

[B25] Elliot A. J. McGregor H. A. (2001). A 2× 2 achievement goal framework. *J. Pers. Soc. Psychol.* 80 501–519. 10.1037/0022-3514.80.3.501 11300582

[B26] Festinger L. (1954). A theory of social comparison processes. *Hum. Relat.* 7 117–140. 10.1177/001872675400700202

[B27] Harackiewicz J. M. Barron K. E. Carter S. M. Lehto A. T. Elliot A. J. (1997). Predictors and consequences of achievement goals in the college classroom: maintaining interest and making the grade. *J. Pers. Soc. Psychol.* 73 1284–1295. 10.1037/0022-3514.73.6.1284

[B28] Harackiewicz J. M. Barron K. E. Elliot A. J. (1998). Rethinking achievement goals: When are they adaptive for college students and why? *Educ. Psychol.* 33 1–21. 10.1207/s15326985ep3301_1

[B29] Harackiewicz J. M. Barron K. E. Pintrich P. R. Elliot A. J. Thrash T. M. (2002). Revision of achievement goal theory: necessary and illuminating. *J. Educ. Psychol.* 94 638–645. 10.1037/0022-0663.94.3.638

[B30] Harackiewicz J. M. Barron K. E. Tauer J. M. Carter S. M. Elliot A. J. (2000). Short-term and long-term consequences of achievement goals: predicting interest and performance over time. *J. Educ. Psychol.* 92 316–330. 10.1037/0022-0663.92.2.316

[B31] Hayes A. F. (2013). *Introduction to Mediation, Moderation and Conditional Process Analysis: A Regression-Based Approach.* New York, NY: Guilford Press.

[B32] Hayes A. F. (2015). An index and test of linear moderated mediation. *Multivariate Behav. Res.* 50 1–22. 10.1080/00273171.2014.962683 26609740

[B33] Hidi S. Harackiewicz J. M. (2000). Motivating the academically unmotivated: a critical issue for the 21st century. *Rev. Educ. Res.* 70 151–179. 10.2307/1170660

[B34] Huguet P. Dumas F. Marsh H. Régner I. Wheeler L. Suls J. (2009). Clarifying the role of social comparison in the big-fish–little-pond effect (BFLPE): an integrative study. *J. Pers. Soc. Psychol.* 97 156–170. 10.1037/a0015558 19586246

[B35] Huguet P. Dumas F. Monteil J. M. Genestoux N. (2001). Social comparison choices in the classroom: further evidence for students’ upward comparison tendency and its beneficial impact on performance. *Eur. J. Soc. Psychol.* 31 557–578. 10.1002/ejsp.81

[B36] IBM SPSS (2017). *IBM SPSS Statistics for Windows, Version 25.0.* Armonk, NY: IBM Corp.

[B37] Jose P. E. (2016). The merits of using longitudinal mediation. *Educ. Psychol.* 51 331–341. 10.1080/00461520.2016.1207175

[B38] Marsh H. W. (1987). The big-fish-little-pond effect on academic self-concept. *J. Educ. Psychol.* 79 280–295. 10.1037/0022-0663.79.3.280

[B39] Marsh H. W. (1990). A multidimensional, hierarchical model of self-concept: theoretical and empirical justification. *Educ. Psychol. Rev.* 2 77–172. 10.1007/BF01322177

[B40] Marsh H. W. (1993). The multidimensional structure of academic self-concept: invariance over gender and age. *Educ. Res. J.* 30 841–860. 10.2307/1163206 8235047

[B41] Marsh H. W. Byrne B. M. Shavelson R. J. (1988). A multifaceted academic self-concept: its hierarchical structure and its relation to academic achievement. *J. Educ. Psychol.* 80 366–380. 10.1037/0022-0663.80.3.366

[B42] Marsh H. W. Chessor D. Craven R. Roche L. (1995). The effects of gifted and talented programs on academic self-concept: the big fish strikes again. *Am. Educ. Res. J.* 32 285–319. 10.2307/1163433

[B43] Marsh H. W. Kuyper H. Morin A. J. Parker P. D. Seaton M. (2014a). Big-fish-little-pond social comparison and local dominance effects: integrating new statistical models, methodology, design, theory and substantive implications. *Learn. Instr.* 33 50–66. 10.1016/j.learninstruc.2014.04.002

[B44] Marsh H. W. Kuyper H. Seaton M. Parker P. D. Morin A. J. Möller J. (2014b). Dimensional comparison theory: an extension of the internal/external frame of reference effect on academic self-concept formation. *Contemp. Educ. Psychol.* 39 326–341. 10.1016/j.cedpsych.2014.08.003

[B45] Marsh H. W. Parker J. (1984). Determinants of student self-concept: Is it better to be a relatively large fish in a small pond even if you don’t learn to swim as well? *J. Pers. Soc. Psychol.* 47 213–231. 10.1037/0022-3514.47.1.213

[B46] Marsh H. W. Seaton M. (2015). “The big-fish-little-pond effect, competence self-perceptions, and relativity: substantive advances and methodological innovation,” in *Advances in Motivation Science*, Vol. 2 ed. Elliott A. J. (New York, NY: Elsevier), 127–184. 10.1016/bs.adms.2015.05.002

[B47] Marsh H. W. Trautwein U. Lüdtke O. Baumert J. Köller O. (2007). The big-fish-little-pond effect: persistent negative effects of selective high schools on self-concept after graduation. *Am. Educ. Res. J.* 44 631–669. 10.3102/0002831207306728

[B48] Marsh H. W. Xu K. M. Parker P. D. Hau K. T. Pekrun R. Elliot A. (2021). Moderation of the big-fish-little-pond effect: juxtaposition of evolutionary (darwinian-economic) and achievement motivation theory predictions based on a delphi approach. *Educ. Psychol. Rev.* 33 1353–1378. 10.1007/s10648-020-09583-5

[B49] Marsh H. W. Yeung A. S. (1997). Coursework selection: the effects of academic self-concept and achievement. *Am. Educ. Res. J.* 34 691–720. 10.2307/1163354

[B50] Maxwell S. E. Cole D. A. Mitchell M. A. (2011). Bias in cross-sectional analyses of longitudinal mediation: partial and complete mediation under an autoregressive model. *Multivariate Behav. Res.* 46 816–841. 10.1080/00273171.2011.606716 26736047

[B51] Middleton M. J. Midgley C. (1997). Avoiding the demonstration of lack of ability: an underexplored aspect of goal theory. *J. Educ. Psychol.* 89 710–718. 10.1037/0022-0663.89.4.710

[B52] Midgley C. Kaplan A. Middleton M. (2001). Performance-approach goals: good for what, for whom, under what circumstances, and at what cost? *J. Educ. Psychol.* 93 77–86. 10.1037/0022-0663.93.1.77

[B53] Mouratidis A. Michou A. Demircioğlu A. N. Sayil M. (2018). Different goals, different pathways to success: performance-approach goals as direct and mastery-approach goals as indirect predictors of grades in mathematics. *Learn. Individ. Differ.* 61 127–135. 10.1016/j.lindif.2017.11.017

[B54] Muthén L. K. Muthén B. O. (2017). *Mplus user’s Guide*, 8th Edn. Los Angeles, CA: Muthén & Muthén.

[B55] Nagengast B. Marsh H. W. (2012). Big fish in little ponds aspire more: mediation and cross-cultural generalizability of school-average ability effects on self-concept and career aspirations in science. *J. Educ. Psychol.* 104 1033–1053. 10.1037/a0027697

[B56] Nicholls J. G. (1984). Achievement motivation: conceptions of ability, subjective experience, task choice, and performance. *Psychol. Rev.* 91 328–346. 10.1037/0033-295X.91.3.328

[B57] OECD (2013). *PISA 2012 Results: Ready to Learn: Students’ Engagement, Drive and Self-Beliefs*, Vol. 3. Paris: PISA, OECD Publishing.

[B58] OECD (2017). *PISA 2015 Results: Students’ Well-Being*, Vol. 3. Paris: PISA, OECD Publishing.

[B59] OECD (2018). *The Resilience of Students with an Immigrant Background: Factors that Shape Well-Being.* Paris: OECD Publishing.

[B60] Parker P. D. Marsh H. W. Ciarrochi J. Marshall S. Abduljabbar A. S. (2014). Juxtaposing math self-efficacy and self-concept as predictors of long-term achievement outcomes. *Educ. Psychol.* 34 29–48. 10.1080/01443410.2013.797339

[B61] Pintrich P. R. (2000a). “The role of goal orientation in self-regulated learning,” in *Handbook of Self-Regulation*, eds Boekaerts M. Pintrich P. R. Zeidner M. (San Diego, CA: Academic Press), 451–502. 10.1016/B978-012109890-2/50043-3

[B62] Pintrich P. R. (2000b). An achievement goal theory perspective on issues in motivation terminology, theory, and research. *Contemp. Educ. Psychol.* 25 92–104. 10.1006/ceps.1999.1017 10620384

[B63] Pintrich P. R. (2000c). Multiple goals, multiple pathways: the role of goal orientation in learning and achievement. *J. Educ. Psychol.* 92 544–555. 10.1037/0022-0663.92.3.544

[B64] Pomerantz E. M. Ruble D. N. Frey K. S. Greulich F. (1995). Meeting goals and confronting conflict: children’s changing perceptions of social comparison. *Child Dev.* 66 723–738. 10.2307/1131946 7789198

[B65] Régner I. Escribe C. Dupeyrat C. (2007). Evidence of social comparison in mastery goals in natural academic settings. *J. Educ. Psychol.* 99 575–583. 10.1037/0022-0663.99.3.575

[B66] Scherrer V. Preckel F. Schmidt I. Elliot A. J. (2020). Development of achievement goals and their relation to academic interest and achievement in adolescence: a review of the literature and two longitudinal studies. *Dev. Psychol.* 56 795–814. 10.1037/dev0000898 32052983

[B67] Schreiber J. B. Nora A. Stage F. K. Barlow E. A. King J. (2006). Reporting structural equation modeling and confirmatory factor analysis results: a review. *J. Educ. Res.* 99 323–338. 10.3200/JOER.99.6.323-338

[B68] Seaton M. (2007). *The Big-Fish-Little-Pond Effect Under the Grill: Tests of its Universality, a Search for Moderators, and the Role of Social Comparison.* Doctoral dissertation. Glasgow: Glasgow University

[B69] Seaton M. Marsh H. W. Craven R. G. (2010). Big-fish-little-pond effect: generalizability and moderation—Two sides of the same coin. *Am. Educ. Res. J.* 47 390–433. 10.3102/0002831209350493

[B70] Seaton M. Marsh H. W. Yeung A. S. Craven R. (2011). The big fish down under: examining moderators of the ‘big-fish-little-pond’effect for Australia’s high achievers. *Aust. J. Educ.* 55 93–114. 10.1177/000494411105500202

[B71] Senko C. (2019). When do mastery and performance goals facilitate academic achievement? *Contemp. Educ. Psychol.* 59:101795. 10.1016/j.cedpsych.2019.101795

[B72] Senko C. Dawson B. (2017). Do normative-based and appearance-based performance goals have different effects? A meta-analysis. *J. Educ. Psychol.* 109 574–589.

[B73] Senko C. Durik A. M. Patel L. Lovejoy C. M. Valentiner D. (2013). Performance-approach goal effects on achievement under low versus high challenge conditions. *Learn. Instr.* 23 60–68. 10.1016/j.learninstruc.2012.05.006

[B74] Senko C. Freund A. M. (2015). Are mastery-avoidance achievement goals always detrimental? An adult development perspective. *Motivat. Emot.* 39 477–488. 10.1007/s11031-015-9474-1

[B75] Senko C. Tropiano K. L. (2016). Comparing three models of achievement goals: goal orientations, goal standards, and goal complexes. *J. Educ. Psychol.* 108 1178–1192. 10.1037/edu0000114

[B76] Skaalvik E. M. (1997). Self-enhancing and self-defeating ego orientation: relations with task and avoidance orientation, achievement, self-perceptions, and anxiety. *J. Educ. Psychol.* 89 71–81. 10.1037/0022-0663.89.1.71

[B77] Spector P. E. (2019). Do not cross me: optimizing the use of cross-sectional designs. *J. Bus. Psychol.* 34 125–137. 10.1007/s10869-018-09613-8

[B78] Thijs J. Verkuyten M. Helmond P. (2010). A further examination of the big-fish–little-pond effect: perceived position in class, class size, and gender comparisons. *Soc. Educ.* 83 333–345. 10.1177/0038040710383521

[B79] Urdan T. Kaplan A. (2020). The origins, evolution, and future directions of achievement goal theory. *Contemp. Educ. Psychol.* 61:101862. 10.1016/j.cedpsych.2020.101862

[B80] Van Yperen N. W. Blaga M. Postmes T. (2015). A meta-analysis of the impact of situationally induced achievement goals on task performance. *Hum. Perform.* 28 165–182. 10.1080/08959285.2015.1006772

[B81] Van Yperen N. W. Elliot A. J. Anseel F. (2009). The influence of mastery-avoidance goals on performance improvement. *Eur. J. Soc. Psychol.* 39 932–943. 10.1002/ejsp.590

[B82] Walker I. Pettigrew T. F. (1984). Relative deprivation theory: an overview and conceptual critique. *Br. J. Soc. Psychol.* 23 301–310. 10.1111/j.2044-8309.1984.tb00645.x

[B83] Wang C. J. Biddle S. J. Elliot A. J. (2007). The 2× 2 achievement goal framework in a physical education context. *Psychol. Sport Exerc.* 8 147–168. 10.1016/j.psychsport.2005.08.012

[B84] Wang Z. (2015). Examining big-fish-little-pond-effects across 49 countries: a multilevel latent variable modelling approach. *Educ. Psychol.* 35 228–251. 10.1080/01443410.2013.827155

[B85] Wang Z. Bergin D. A. (2017). Perceived relative standing and the big-fish-little-pond effect in 59 countries and regions: analysis of TIMSS 2011 data. *Learn. Individ. Differ.* 57 141–156. 10.1016/j.lindif.2017.04.003

[B86] Wouters S. Colpin H. Van Damme J. Verschueren K. (2015). Endorsing achievement goals exacerbates the big-fish-little-pond effect on academic self-concept. *Educ. Psychol.* 35 252–270. 10.1080/01443410.2013.822963

[B87] Zell E. Alicke M. D. (2009). Contextual neglect, self-evaluation, and the frog pond effect. *J. Pers. Soc. Psychol.* 97 467–482. 10.1037/a0015453 19686002

[B88] Zimmerman B. J. Kitsantas A. (1999). Acquiring writing revision skill: shifting from process to outcome self-regulatory goals. *J. Educ. Psychol.* 91 241–250. 10.1037/0022-0663.91.2.241

