# Supplementary material for: Individual Strivings in Social Comparison Processes: Achievement Motivation Goals in the Big-Fish-Little-Pond Effect
Source: Front Psychol. 2022 Apr 18;13:677997. doi: 10.3389/fpsyg.2022.677997 (PMC9062594; doi:10.3389/fpsyg.2022.677997)
Supplement: Supplementary file 1 [file Table_1.docx]

**Appendix A**

Achievement-motivation goals scale—items and standardized factor loadings (CFA).

| **1.** È importante per me essere più bravo dei miei compagni di classe. |
| --- |
| **2.** Per me è importante andare meglio dei miei compagni di classe. |
| **3.** Il mio obiettivo a scuola è di prendere voti migliori rispetto alla maggior parte dei miei compagni di classe. |
| **4.** Qualche volta sono preoccupato che non riuscirò ad imparare tutto ciò che mi viene insegnato a scuola. |
| **5.** Certe volte ho paura di non riuscire a capire bene gli argomenti delle lezioni come vorrei. |
| **6.** Sono sinceramente dispiaciuto quando penso che potrei non riuscire ad imparare tutto quello che c’è da imparare a scuola. |
| **7.** A scuola voglio imparare il più possibile. |
| **8.** È importante che io capisca il contenuto delle lezioni il meglio possibile. |
| **9.** Desidero poter padroneggiare perfettamente le materie che ci spiegano a scuola. |
| **10.** L’importante è evitare di andare peggio a scuola rispetto agli altri compagni di classe. |
| **11.** Il mio obiettivo è di evitare di ritrovarmi tra i peggiori studenti della classe. |
| **12.** La mia paura di avere voti peggiori dei miei compagni di classe mi spinge ad impegnarmi. |
