# Supplementary material for: Individual Strivings in Social Comparison Processes: Achievement Motivation Goals in the Big-Fish-Little-Pond Effect
Source: Front Psychol. 2022 Apr 18;13:677997. doi: 10.3389/fpsyg.2022.677997 (PMC9062594; doi:10.3389/fpsyg.2022.677997)
Supplement: Supplementary file 2 [file Table_2.docx]

**Appendix B**

Sample composition—students who filled in the questionnaire in each class.

| ***Class*** | ***Students*** | |
| --- | --- | --- |
|  | ***Number*** | ***Percentage*** |
| 1 | 20/25 | 80.0% |
| 2 | 20/21 | 95.2% |
| 3 | 19/20 | 95% |
| 4 | 16/18 | 88.9% |
| 5 | 17/22 | 77.3% |
| 6 | 16/18 | 88.9% |
| 7 | 23/26 | 88.5% |
| 8 | 27/32 | 84.4% |
| 9 | 13/16 | 81.3% |
| 10 | 20/23 | 86.9% |
| 11 | 17/20 | 85% |
| 12 | 12/18 | 66.7% |
| 13 | 19/26 | 73.1% |
| 14 | 17/17 | 100% |
| 15 | 22/23 | 95.7% |
| 16 | 16/18 | 88.9% |
| 17 | 21/23 | 88.9% |
| 18 | 22/23 | 95.7% |
| 19 | 17/20 | 85% |
| 20 | 21/24 | 87.5% |
| 21 | 24/24 | 100% |
| 22 | 27/27 | 100% |
| 23 | 24/28 | 85.7% |
| 24 | 17/26 | 65.4% |
| 25 | 15/29 | 51.7% |
| 26 | 13/24 | 54.7% |
| 27 | 21/26 | 80.8% |
| 28 | 23/26 | 88.5% |
| 29 | 15/15 | 100% |
| 30 | 16/20 | 80% |
| 31 | 8/17 | 47.1% |
| 32 | 15/15 | 100% |
| 33 | 17/24 | 70.8% |
| 34 | 21/21 | 100% |
| 35 | 15/19 | 79% |
| 36 | 12/16 | 75% |
| 37 | 14/19 | 73.7% |
| 38 | 16/19 | 84.2% |
