# Supplementary material for: Individual Strivings in Social Comparison Processes: Achievement Motivation Goals in the Big-Fish-Little-Pond Effect
Source: Front Psychol. 2022 Apr 18;13:677997. doi: 10.3389/fpsyg.2022.677997 (PMC9062594; doi:10.3389/fpsyg.2022.677997)
Supplement: Supplementary file 3 [file Table_3.docx]

**Appendix C**

Johnson-Neyman technique—conditional effects of perceived relative position (R-pos) on better future at different values of performance-approach (Perf-Ap).

| ***Perf-Ap*** | ***Effect*** | ***SE*** | ***t*** | ***p*** | ***LLCI*** | ***ULCI*** |
| --- | --- | --- | --- | --- | --- | --- |
| -1.124 | .741 | .042 | 17.502 | .000 | .658 | .824 |
| -.932 | .729 | .039 | 18.632 | .000 | .652 | .805 |
| -.739 | .716 | .036 | 19.665 | .000 | .644 | .787 |
| -.547 | .703 | .034 | 20.450 | .000 | .636 | .771 |
| -.355 | .691 | .033 | 20.816 | .000 | .626 | .756 |
| -.163 | .678 | .033 | 20.633 | .000 | .614 | .743 |
| .029 | .665 | .033 | 19.885 | .000 | .600 | .731 |
| .221 | .653 | .035 | 18.686 | .000 | .584 | .721 |
| .413 | .640 | .037 | 17.221 | .000 | .567 | .713 |
| .606 | .628 | .040 | 15.668 | .000 | .549 | .706 |
| .798 | .625 | .043 | 14.155 | .000 | .530 | .700 |
| .990 | .602 | .047 | 12.751 | .000 | .510 | .695 |
| 1.182 | .590 | .051 | 11.486 | .000 | .489 | .691 |
| 1.374 | .577 | .056 | 10.362 | .000 | .468 | .687 |
| 1.566 | .565 | .060 | 9.372 | .000 | .446 | .683 |
| 1.758 | .552 | .065 | 8.500 | .000 | .424 | .679 |
| 1.951 | .539 | .070 | 7.733 | .000 | .402 | .676 |
| 2.143 | .527 | .075 | 7.055 | .000 | .380 | .673 |
| 2.335 | .514 | .080 | 6.455 | .000 | .358 | .671 |
| 2.527 | .502 | .085 | 5.921 | .000 | .335 | .668 |
| 2.719 | .489 | .090 | 5.444 | .000 | .313 | .665 |
