# Supplementary material for: Individual Strivings in Social Comparison Processes: Achievement Motivation Goals in the Big-Fish-Little-Pond Effect
Source: Front Psychol. 2022 Apr 18;13:677997. doi: 10.3389/fpsyg.2022.677997 (PMC9062594; doi:10.3389/fpsyg.2022.677997)
Supplement: Supplementary file 4 [file Table_4.docx]

**Appendix D**

Johnson-Neyman technique—conditional effects of perceived relative position (R-pos) on better future at different values of performance-avoidance (Perf-Av).

| ***Perf-Av*** | ***Effect*** | ***SE*** | ***t*** | ***p*** | ***LLCI*** | ***ULCI*** |
| --- | --- | --- | --- | --- | --- | --- |
| -1.291 | .790 | .044 | 17.824 | .000 | .703 | .877 |
| -1.112 | .775 | .041 | 18.919 | .000 | .694 | .855 |
| -.933 | .759 | .038 | 20.029 | .000 | .685 | .834 |
| -.755 | .744 | .035 | 21.072 | .000 | .674 | .818 |
| -.576 | .728 | .033 | 21.924 | .000 | .663 | .793 |
| -.397 | .713 | .032 | 22.429 | .000 | .650 | .775 |
| -.218 | .697 | .031 | 22.446 | .000 | .636 | .758 |
| -.040 | .682 | .031 | 21.902 | .000 | .621 | .743 |
| .139 | .666 | .032 | 20.845 | .000 | .603 | .729 |
| .318 | .651 | .034 | 19.418 | .000 | .585 | .716 |
| .496 | .635 | .036 | 17.802 | .000 | .565 | .705 |
| .675 | .620 | .038 | 16.151 | .000 | .544 | .695 |
| .854 | .604 | .041 | 14.568 | .000 | .523 | .686 |
| 1.033 | .589 | .045 | 13.109 | .000 | .500 | .677 |
| 1.211 | .573 | .049 | 11.794 | .000 | .478 | .669 |
| 1.390 | .558 | .052 | 10.623 | .000 | .455 | .661 |
| 1.569 | .542 | .057 | 9.586 | .000 | .431 | .653 |
| 1.747 | .527 | .061 | 8.669 | .000 | .407 | .646 |
| 1.926 | .511 | .065 | 7.858 | .000 | .383 | .639 |
| 2.105 | .496 | .069 | 7.139 | .000 | .359 | .632 |
| 2.284 | .480 | .074 | 6.499 | .000 | .335 | .625 |
